# Bi-layer carbon design for microparticulate silicon anodes

**DOI:** 10.1093/nsr/nwab057

**Published:** 2021-04-06

**Authors:** Weiran Zhang, Sufu Liu, Chunsheng Wang

**Affiliations:** Department of Chemical and Biomolecular Engineering, University of Maryland, USA; Department of Chemical and Biomolecular Engineering, University of Maryland, USA; Department of Chemical and Biomolecular Engineering, University of Maryland, USA

The silicon (3560 mAh/g) anode delivers a 10-times-higher specific capacity than that of the graphite counterpart (372 mAh/g). However, the large volume change of Si during lithiation and de-lithiation processes always result in poor cycling stability. Lots of studies have been focused on the nanoengineering of Si [1], but the high cost, complex fabrication, poor initial coulombic efficiency (iCE) and calendar life limit its commercialization. Microparticulate Si (SiMP) provides a low-cost choice, and its lower surface area enables a higher iCE in principle due to its lower reaction with electrolyte [2]. However, the unavoidable particle pulverization of SiMP cracks the organic-inorganic solid electrolyte interphase (SEI) formed in commercial carbonate electrolytes due to the strong bonding between organic-inorganic SEI and Si, resulting in the formation of a new SEI between pulverized Si particles. The cracking/reforming of SEI isolates the pulverized Si particles and consumes electrolytes and Li stored in the cathode, resulting in a quick capacity decay.

Stabilization of the SEI on SiMP is critical for success of SiMP anodes. We stabilized an LiF-rich SEI on SiMP anode by designing a 2.0 M LiPF_6_ in mixTHF (1 : 1 v/v THF : mTHF) electrolyte. Since LiF SEI has a high interfacial energy (low adhesion) with Li_x_Si/Si, the LiF-rich SEI remained stable during the volume expansion of Si and thus prevented the electrolyte penetration into the pulverized SiMP [3]. Another effective strategy is to use an electronic conductive carbon cage on SiMP to stabilize SEI, which blocks electrolyte penetration and allows the expansion and shrinkage of SiMP within the cage [4]. However, it is quite challenging for the carbon cage to be both mechanically strong and ductile to accommodate the compression during fabrication and the high-volume change without cracking the SEI during cycling [5]. Recently, Yang and co-workers reported a 2-fold buffering strategy to stabilize the carbon cage and SEI by forming a strong graphitic carbon and ductile graphene bi-layer cage on SiMP, which is a milestone in the advancement of SiMP anodes [6].

**Figure 1.**
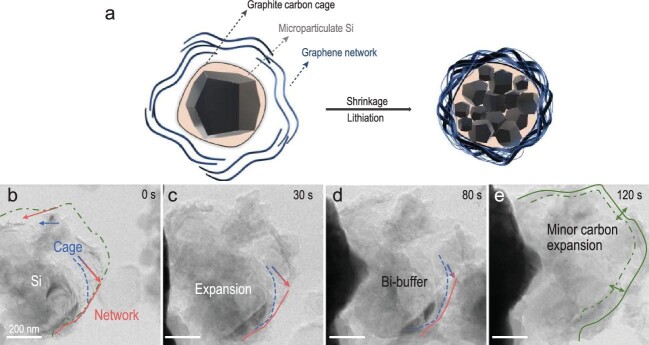
(a) The structure design principle of the microparticulate silicon anode with carbon cage and graphene network. (b–e) *In situ* TEM observation of the lithiation of SiMP@C-GN [6].

Firstly, the carbon cage with a void inside was prepared by chemical vapor deposition (CVD) and alkaline etching, which helps to accommodate the volume expansion of Si particles and suppress its side reaction with electrolytes. Furthermore, to achieve a dense graphene network tightly adhered on the surface of carbon cages, a graphene hydrogel and capillary drying technology were developed. Due to the strong capillary force exerted by the surface tension of water, the graphene nanosheets shrink into a dense monolith and stick on the surface of the carbon cages (SiMP@C-GN), thus enabling a mechanically strong and electrically conductive network (Fig. 1a).

The high mechanical stability of the SiMP@C-GN anode was demonstrated by *insitu* transmission electron microscopy (TEM) observations (Fig. 1b–e). Owing to the high capillary shrinkage and densification, the crosslinked graphene on the carbon cage enables interlayer sliding and stress dissipation to avoid cracking of the carbon cage, which stabilizes the SEI formed, while the voids inside the carbon cage allow Si to have the volume expansion/shrinkage with minimized stress to the graphene/carbon cage. As a result, this designed Si anode demonstrates a high average coulombic efficiency (CE) of  99.5%. A high-capacity retention (>70%) for 500 cycles in the half cell is achieved (∼1.5 mAh/cm^2^), which is much better than that of the SiMP anode with carbon coating only (63% after 100 cycles). Moreover, pouch full cell based on the designed Si anode and LiNi_0.8_Mn_0.1_Co_0.1_O_2_ (NMC811) cathode demonstrated an energy density of 1048 Wh/L after 50 cycles, which is one of the highest values reported for lithium-ion batteries.

The work by Yang and co-workers provides an ingenious structure design where shrunken graphene networks compactly interweave the CVD carbon with preserved voids for SiMP. The bi-layer graphene/carbon cage experiences minimized size change during alloying/de-alloying of SiMP due to its high mechanical strength and the voids inside the cage, which stabilize the SEI. It is worth noting that the tough but ductile bi-layer carbon could satisfy the stringent mechanical requirements for an ‘internal’ buffer and ‘external’ compression in practical fabrication and working conditions. Using high-voltage carbonate electrolytes to match high-energy NMC cathodes, the designed SiMP anodes could remarkably increase the energy density of Li-ion batteries. To commercialize SiMP@C-GN anodes, however, a cheaper synthesizing process to replace the CVD process is desired. The structure of SiMP with a graphene/carbon cage also needs to be optimized to further enhance the initial CE (71%) of SiMP@C-GN and cycling CE when compared with the graphite anode.

***Conflict of interest statement*.** None declared.
